# Trade-off between offspring mass and number: the lightest offspring bear the costs

**DOI:** 10.1098/rsbl.2019.0707

**Published:** 2020-02-12

**Authors:** Joanie Van de Walle, Andreas Zedrosser, Jon E. Swenson, Fanie Pelletier

**Affiliations:** 1 Département de biologie and Centre for Northern Studies, Université de Sherbrooke, Sherbrooke, Québec, Canada J1K 2R1; 2 Department of Natural Sciences and Environmental Health, University of South-Eastern Norway, 3800 Bø i Telemark, Norway; 3 Institute of Wildlife Biology and Game Management, University of Natural Resources and Life Sciences, 1180 Vienna, Austria; 4 Faculty of Environmental Sciences and Natural Resource Management, Norwegian University of Life Sciences, 1432 Ås, Norway

**Keywords:** life-history trade-offs, individual heterogeneity, litter size, offspring mass

## Abstract

Life-history theory predicts a trade-off between offspring size and number. However, the role of intra-litter phenotypic variation in shaping this trade-off is often disregarded. We compared the strength of the relationship between litter size and mass from the perspective of the lightest and the heaviest yearling offspring in 110 brown bear litters in Sweden. We showed that the mass of the lightest yearlings decreased with increasing litter size, but that the mass of the heaviest yearling remained stable, regardless of litter size. Consistent with a conservative reproductive strategy, our results suggest that mothers maintained a stable investment in a fraction of the litter, while transferring the costs of larger litter size to the remaining offspring. Ignoring intra-litter phenotypic variation may obscure our ability to detect a trade-off between offspring size and number.

## 1. Introduction

Under limited resources, parents must make decisions regarding energy allocation to reproduction, implying reduced allocation to other functions [1,2]. Species producing multiple offspring simultaneously face an additional dilemma; invest in either a few large or several small, offspring [3]. Evidence of the trade-off between offspring size and number at the interspecific level abounds [2,4]. Opposing results are found at the intraspecific level [5], but accounting for the masking effects of environmental conditions and individual heterogeneity [6] has helped clarify this trade-off in several species [7,8].

Many studies investigating trade-offs between offspring mass and number consider the average effect of litter/clutch size [9–13] assuming that, in a given environment, parents should produce an optimal number of offspring [9] and allocate resources equally among them [3]. This assumption is challenged, however, by empirical observations showing large within litter/clutch variation in offspring mass [14]. Reasons for such variation are still poorly understood, but several explanations have been proposed. For example, mothers may be unable to provision their offspring equally, especially younger mothers in adverse environmental conditions [15]. Further, offspring can actively influence how much energy is directed towards them, through differential solicitation and sibling competition, causing variation in sibling mass [16,17].

Maternal effects and strategies can also generate phenotypic variation among offspring. Diversified bet-hedging (i.e. diversification of offspring phenotypes [18]) may be a female strategy to minimize between-year variation in reproductive success under unpredictable environmental conditions [14,18], although its adaptive significance and occurrence in nature remain unclear [15,19]. In egg-producing species, asynchronous hatching can create intra-clutch phenotypic variation [20], and mothers can adjust investment differentially following egg order [21]. In many bird species, mothers produce a caste of larger (core) and a caste of smaller (marginal), expendable nestlings within the same clutch [22]. Whether adaptive or not, large within litter/clutch individual phenotypic heterogeneity suggests that the trade-off between offspring mass and number may be borne differently by offspring from the same mother.

We investigated the trade-off between offspring mass and number in brown bears (*Ursus arctos*) and compared its importance for the lightest and the heaviest yearlings in a litter. Brown bear mothers give birth to 1–4 cubs during hibernation. Cubs separate from their mother after den emergence in their second or third spring in Scandinavia [23]. Although knowledge of the relative and temporal contribution of milk in cubs' diet is limited, cubs start feeding on solid food in their first summer, but continue to nurse throughout summer and autumn [24], probably also after entering the den. Thus, maternal milk may represent the most important food source for cubs in their first year. Previous studies have shown that yearling mass decreases with litter size [12,25] and is more variable in larger litters [25], suggesting heterogeneity in the response of individual yearling mass to litter size. By adding 8 years of recently collected data, we expected to confirm the negative relationship between yearling mass and number. Then, we predicted a stronger negative relationship between yearling mass and litter size when investigated from the perspective of the lightest compared to the heaviest yearling, suggesting that smaller yearlings bear the energetic cost of the trade-off between offspring mass and number.

## 2. Material and methods

We used data on brown bear family groups collected during captures from a helicopter by darting conducted in late April–early May in south-central Sweden, 1990–2016. For further details on bear captures see Arnemo *et al*. [26]. Females were equipped with VHF (before 2003) or VHF/GPS (after 2003) collars. For ethical reasons, only family groups with yearlings (approx. 15 months old), not cubs-of-the-year (hereafter ‘cubs’), were captured. Upon capture, we determined yearling sex, and weighed all bears with a spring scale. As a surrogate for maternal size, head circumference (cm), reflecting skeletal size [10,25], was measured at the widest part of the zygomatic arch between eyes and ears with a tape. Because bears were usually captured within two weeks [25], yearling mass was not adjusted for capture date. Age of mothers followed since birth (54%) was known; a premolar tooth was extracted for age determination [27] for others. Although not captured, mothers with cubs were located and cubs counted from the ground or a helicopter at least three times annually. Because yearling mass can increase after partial litter loss [12], we only considered litters with no pre-capture loss (73% of litters).

Population density commonly affects body mass in large mammals [28], such as the brown bear [25]. Thus, we calculated a relative index of local population density for each family group during the yearling year by extracting a weighted mean of local density within a circular buffer of 7.16 km (average home range radius for an adult female with yearlings [29]) around the median of bear locations using VHF data prior to 2003 and a combination of VHF and GPS data from 2003 onward. Annual maps of bear density were constructed using scat-derived DNA collections conducted at the county level in specific years [30], corrected for annual trends using country-wide sightings of bears in the autumn through the Swedish Large Carnivore Observation Index [31]. See electronic supplementary materials (appendix S1) for more details on density estimation.

First, we tested for the presence of a trade-off between offspring mass and number (average effect) using linear mixed effect models, with yearling body mass (log-transformed to meet the model assumption of homoscedasticity [32]) as response variable. Litter size (set as continuous [25]) and other variables known or expected to affect yearling mass, i.e. maternal size, offspring sex, litter sex ratio and population density index [12,25], were included as fixed effects. To account for the potential masking effect of female size on life-history trade-offs [6,33], we included an interaction between maternal size and litter size. Random effects included year of capture and litter identity nested in maternal identity.

Second, we compared the importance of the trade-off, i.e. the slope of the relationship, between yearling mass and number for only the lightest and heaviest yearlings within each litter (rank effect). To provide a baseline for statistical comparison with larger litters, singletons were also included and randomly attributed a rank (‘lightest’ or ‘heaviest’). See electronic supplementary materials (appendix S2) for more details on the yearling classification procedure. We used linear mixed effects models with log-transformed yearling mass of only the lightest and heaviest yearlings for each litter as the response variable and the same fixed and random effects structure as above, but we added an interaction term between litter size and yearling rank (2 levels: ‘lightest’, ‘heaviest’). For both analyses (‘average effect’ and ‘rank effect’), the starting models included all fixed effects and we obtained the final models by backwards selection using Likelihood ratio tests. Variance inflation factors were all less than 3. Effect sizes are presented as means of per cent mass change per additional yearling in a litter with 95% confidence intervals calculated as exponentiated model coefficients subtracted by 1. All analyses were performed using R v. 3.4.3 [34].

## 3. Results

**Figure 1.**
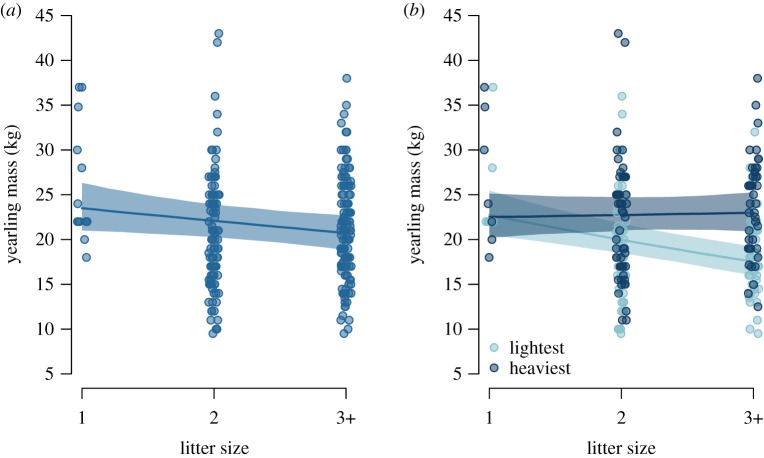
Trade-off between offspring mass and number in brown bears from Sweden, 1990–2016, investigated using (*a*) all and (*b*) only the lightest (light blue) and heaviest (dark blue) yearlings from a litter. Represented are observations (circles) with model predictions (lines) and 95% confidence intervals (polygons) back-transformed to the original scale for males (*a*) or both sexes (*b*) from average-sized mothers (*a,b*) and at average population density (*a,b*).

We obtained data from 110 litters without litter reduction from 54 mothers (maximum 5 litters/mother), totalling 250 yearlings (122 females, 128 males) [35]. Litter size varied from 1 to 4 (1: *n* = 14, 2: *n* = 54, 3: *n* = 40, 4: *n* = 2). Due to few litters of 4, we combined litter sizes 3 and 4. There was no interactive effect of litter size and maternal size on yearling mass (table 1*a*). Yearling mass was unaffected by maternal age and litter sex ratio, but yearlings were heavier if they were males, their mother was larger and population density was lower. There was a significant negative relationship between yearling mass and litter size, revealing a trade-off between offspring mass and number in the population (figure 1*a*); yearling mass decreased by 6.1% (95% CI = [−11.0%, −0.8%]) per additional yearling in a litter.

**Table 1.** Final models obtained by backward selection to test for the presence of a trade-off between offspring mass and number (*a*, average effect) and whether the trade-off was borne differently by the ‘lightest’ and ‘heaviest’ yearlings in a litter (*b,* rank effect) in brown bears in Sweden, 1990–2016.

				95% CI				
variables	*β*	s.e.	*t*-value	lower	upper		variance	s.d.
(*a*) *log(yearling mass)* − *average effect (conditional R^2^ = 77%, marginal R^2^ = 24%)*^a^								
fixed effects						random effects		
intercept	0.87	0.38	2.32	0.14	1.61	litter ID × maternal ID	0.01	0.11
sex (male)	0.06	0.02	2.58	0.01	0.10	maternal ID	0.01	0.08
litter size	−0.06	0.03	−2.28	−0.12	−0.01	year	0.03	0.17
maternal size	0.04	0.01	6.36	0.03	0.05	residual	0.02	0.14
local density	−0.26	0.10	−2.57	−0.47	−0.06			
*variables excluded: litter size × maternal size (χ*^2^ = *0.08, p = 0.78), maternal age (χ*^2^ = *0.98, p = 0.32), sex ratio (χ*^2^ = *2.52, p = 0.11)*								
(*b*) *log(yearling mass) − rank effect (conditional R^2^ = 91%, marginal R^2^ = 33%)*								
fixed effects						random effects		
intercept	0.77	0.37	2.05	0.04	1.49	litter ID × maternal ID	0.02	0.13
litter size	0.01	0.03	0.35	−0.05	0.07	maternal ID	0.01	0.07
maternal size	0.04	0.01	6.65	0.03	0.05	year	0.03	0.18
local density	−0.23	0.10	−2.35	−0.43	−0.04	residual	0.01	0.09
rank (lightest)	0.15	0.06	2.44	0.03	0.28			
litter size *×* rank	−0.14	0.03	−5.56	−0.19	−0.09			
*variables excluded: litter size × maternal size (χ*^2^ = *0.06, p = 0.81), sex (χ*^2^ = *0.00, p = 0.99), sex ratio (χ*^2^ *= 0.00, p = 0.96), maternal age (χ*^2^ *= 0.85, p = 0.36)*								

^a^Marginal and conditional *R^2^* refer to the variance explained by fixed effects alone and both fixed and random effects, respectively [36].

We ranked 206 yearlings as ‘lightest’ or ‘heaviest’. In litters of 3–4, yearlings with intermediate mass (*n* = 44) were removed from further analyses. Within a given litter, the lightest yearlings were more often females (61%) and the heaviest were more often males (66%). Observed sex-, litter size- and rank-specific yearling masses are presented in the electronic supplementary materials (appendix S3). We found no interactive effect of litter size and maternal size on the mass of the lightest and heaviest yearlings (table 1*b*). The mass of lightest and heaviest yearlings was affected by maternal size and population density, but not by sex, litter sex ratio or maternal age. However, the slope of the relationship between yearling size and litter size differed by yearling rank, i.e. there was a significant interaction between offspring rank and litter size. The lightest yearlings were affected by litter size, as mass of the lightest yearling decreased by 12.2% (95% CI = [−21.1%, −2.3%]) per additional yearling in a litter (figure 1*b*). However, the mass of the heaviest yearling was unaffected (per cent mass change: 1.0%, 95% CI = [−4.6, 7.0]) by litter size (figure 1*b*).

## 4. Discussion

Based on life-history theory, offspring mass should decline as the number of offspring increases [2]. However, large intra-litter variation in offspring mass suggests heterogeneity in the response of individual offspring to increasing litter size. Our objective was to contrast the strength of the trade-off among offspring from the same litter. We found a non-homogeneous trade-off between yearling mass and number. Indeed, the mass of the lightest yearling in a litter declined with litter size, whereas the mass of the heaviest yearling remained stable, regardless of litter size. Similar results were found using only litter sizes of 2 and 3–4 (electronic supplementary materials; appendix S4), thus reinforcing our conclusion. Our results suggest that smaller offspring bear the cost of the trade-off between offspring mass and number in brown bears.

The heaviest yearlings in litters greater than or equal to 2 were as heavy as singletons, suggesting that mothers allocate resource disproportionately among offspring regardless of litter size, raising at least one heavy yearling. In birds, parents often ‘play favourite’, preferentially directing energy towards some nestlings and only ‘surplus’ energy (if any) to others [22]. In ungulates producing singletons, mothers usually follow a conservative tactic, transferring the cost of current reproduction to subsequent offspring [33,37] through reduced mass gain and survival [33]. Further, despite the absence of an absolute sex-related mass difference between heaviest and lightest yearlings, the heaviest yearlings within a given litter were mostly males. Sons often receive biased maternal allocation, compared to daughters, in polygynous species exhibiting sexual dimorphism [38] to improve their future reproductive success [39]. Biased allocation towards males can increase the energetic costs of reproduction when producing male offspring [40–42], which might be transferred to litter mates, as reflected by the lower mass of the lightest yearlings within a litter, especially in larger litters.

Brown bear cubs nurse throughout their first year [24], suggesting that differential maternal provisioning should persist to yearling age. Our study includes yearlings consuming solid food, however, its relative contribution to the diet is unknown. Access to food resources (mainly berries in autumn [43]), and thus foraging opportunities, should be similar among litter mates, although scramble competition or compensatory feeding is possible [44]. Evaluating the persistence of the trade-off over developmental stages and relative contribution of maternal provisioning would help determine the role of maternal effects in brown bears, although ethical considerations made this impossible here. The persistence of the trade-off between offspring mass and number may vary among species with different feeding strategies and parental control over food access.

Life-history trade-offs can be masked by environmental conditions and individual differences in energy allocation [6], and costs of reproduction are difficult to detect empirically [45]. We show the potential for between-offspring individual variation to obscure the trade-off between offspring mass and number. Focusing on mean offspring mass may hinder our ability to detect, or cause an underestimation of, the cost of producing larger litters, which may be borne by only a fraction of the offspring. This could explain, in part, why a large number of empirical studies have failed to detect this trade-off [46]. Mass at independence can affect an individual's future survival and reproductive success [47,48] (but see [49]). Being born into a large litter may thus have long-term fitness consequences for offspring, depending on their relative size, especially for female brown bears, as lifetime reproductive success is determined by their mass as yearlings [50]. From a mother's perspective, however, favouring one offspring in a large litter may result in a stable fitness return that can be augmented under conditions favouring the fitness of ‘extra’ offspring.

## Supplementary Material

Appendices S1-S4

## Data accessibility

Data are available from the Dryad Digital Repository: https://doi.org/10.5061/dryad.b2rbnzs99 [35].
