# Supplementary material for: Trade-off between offspring mass and number: the lightest offspring bear the costs
Source: Biol Lett. 2020 Feb 12;16(2):20190707. doi: 10.1098/rsbl.2019.0707 (PMC7058944; doi:10.1098/rsbl.2019.0707)
Supplement: Appendices S1-S4 [file rsbl20190707supp1.docx]

**Title:** **Trade-off between offspring mass and number: the lightest offspring bear the costs**

**Authors:** Joanie Van de Walle^1^, Andreas Zedrosser^2,3^, Jon E. Swenson^4^, and Fanie Pelletier^1^

**Affiliations:**

^1^Département de biologie & Centre for Northern Studies, Université de Sherbrooke, Sherbrooke, Canada, Québec, Canada J1K 2R1

^2^Department of Natural Sciences and Environmental Health, University of South-Eastern Norway, NO-3800 Bø i Telemark, Norway

^3^Institute of Wildlife Biology and Game Management, University of Natural Resources and Life Sciences, A-1180 Vienna, Austria

^4^Faculty of Environmental Sciences and Natural Resource Management, Norwegian University of Life Sciences, NO-1432 Ås, Norway

*Correspondence to: joanie.van.de.walle@usherbrooke.ca, Tel: +1 (819)-821-8000 #63020, ORCID iD: 0000-0002-5137-1851

SUPPLEMENTARY MATERIALS

**Appendix S1: Bear density estimation**

We calculated a relative index of local population density for each family group during the yearling year. Annual grids (10 x 10 km cells) of bear density were constructed using two sources of information: 1) county-wide scat-derived DNA collections obtained during organized efforts [1] and 2) annual and country-wide sightings of bears in the fall through the Swedish Large Carnivore Observation Index, LCOI [2]. Data from scat collections were used to produce maps of bear density during the years of scat collection and those maps were corrected for annual trends using the LCOI for the period 1998-2015 [3]. For the periods 1990-1998 and 2015-2016, we assumed a stable density [4] and used the 1998 and 2015 grids, respectively. We used the spatio-temporally explicit maps to derive local population density by extracting a weighted mean of local density within a circular buffer of 7.16 km (average home range radius for an adult female with yearlings [5]) around the median of bear localisations using VHF data prior to 2003 and a combination of VHF and GPS data from 2003 onward. To improve accuracy, we only retained GPS fixes with dilution of precision values <10. We only used relocations during the non-denning period, i.e. between 1 May and 1 November [6].

**Appendix S2: Classification procedure for yearlings**

For each litter of 2 to 4 yearlings, we retained only the lightest and the heaviest yearlings. Sometimes, two or more yearlings had the same mass. In those cases, the relative rank of yearlings was attributed randomly. In singleton litters, resources are not shared among siblings and singletons should receive all energy allocated to reproduction by females. Because of this assumption, singleton litters were included in the analyses to provide a baseline for statistically comparing the mass of those singletons to larger litters. To do so, singletons were randomly classified as “lightest” or “heaviest” in the analyses. Randomisation was performed until the number of “lightest” and “heaviest” yearlings from singleton litters was balanced; i.e. because our sample included 14 such litters, we stopped the procedure when 7 singleton yearlings were randomly classified as “lightest” and 7 as “heaviest”.

**Appendix S3: Supplementary tables and figures**

**Table S1.** Observed sex- and litter size-specific mass (kg) of yearling brown bears classified as the “lightest” or “heaviest” in litters from south-central Sweden, 1990-2016.

|  |  | Males | | | Females | | | All | | |
| --- | --- | --- | --- | --- | --- | --- | --- | --- | --- | --- |
| Litter size | Yearling rank | Mean | SD | *n* | Mean | SD | *n* | Mean | SD | *n* |
| 1 | Lightest* | 22.0 | 0.0 | 3 | 27.3 | 7.1 | 4 | 25.0 | 5.7 | 7 |
|  | Heaviest | 28.8 | 7.6 | 5 | 21.0 | 4.2 | 2 | 26.5 | 5.7 | 7 |
|  | All | 26.2 | 6.7 | 8 | 25.2 | 6.6 | 6 | 25.8 | 6.4 | 14 |
| 2 | Lightest | 19.9 | 6.2 | 17 | 19.5 | 5.8 | 37 | 19.6 | 5.9 | 54 |
|  | Heaviest | 23.3 | 7.3 | 34 | 20.4 | 5.2 | 20 | 22.2 | 6.7 | 54 |
|  | All | 22.2 | 7.1 | 51 | 19.8 | 5.5 | 57 | 20.9 | 6.4 | 108 |
| 3 | Lightest | 19.1 | 5.8 | 20 | 17.8 | 3.9 | 22 | 18.4 | 4.8 | 42 |
|  | Heaviest | 23.8 | 5.8 | 29 | 24.3 | 5.3 | 13 | 23.9 | 5.6 | 42 |
|  | All | 21.3 | 5.8 | 69 | 21.0 | 5.5 | 59 | 21.2 | 5.7 | 128 |

*Yearling rank was attributed randomly for singletons.

**Table S2:** Observed litter size-specific sex ratios (proportion of males) among yearling brown bears in south-central Sweden, 1990-2016.

|  | Mean | SD | *n* (litters) |
| --- | --- | --- | --- |
| All yearlings | 0.51 | 0.37 | 110 |
| Litter size = 1 | 0.57 | 0.51 | 14 |
| Litter size = 2 | 0.47 | 0.37 | 54 |
| Litter size = 3&4 | 0.53 | 0.31 | 42 |

**Table S3:** Sex-specific composition of brown bear yearling litters in south-central Sweden, 1990-2016.

| Sex-specific litter composition | *n* (litters) |
| --- | --- |
| M | 8 |
| F | 6 |
| MM | 13 |
| FF | 16 |
| MF | 25 |
| MMM | 6 |
| MMF | 16 |
| MFF | 12 |
| FFF | 6 |
| MMMM | 1 |
| MMMF | 1 |
| MMFF | 0 |
| MFFF | 0 |
| FFFF | 0 |

**
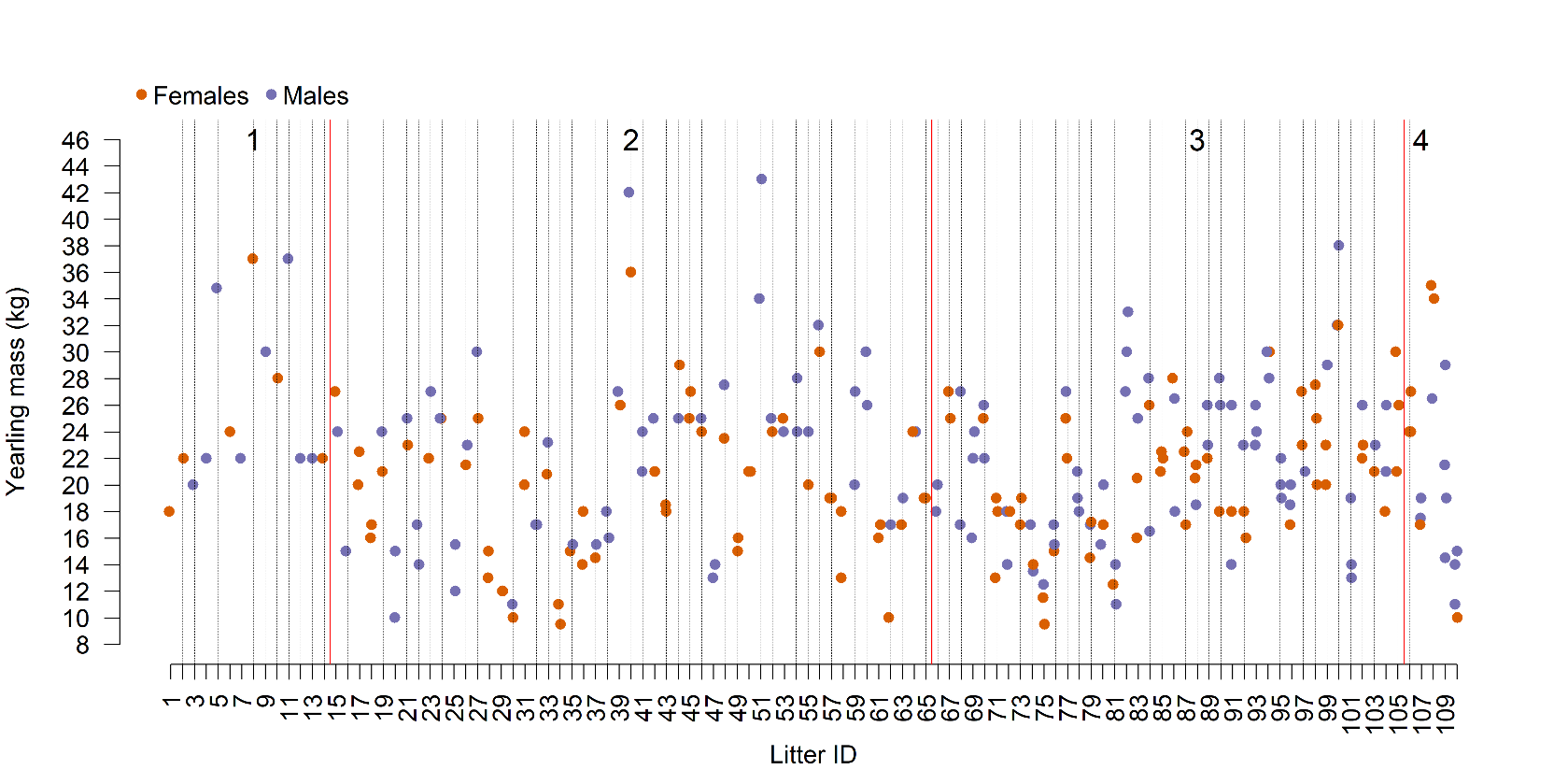
**

**Figure S1:** Observed sex and litter size-specific distribution of yearling mass in brown bear litters in south-central Sweden, 1990-2016. Litter sizes (1, 2, 3 and 4) are shown on top and are separated by red lines.

**Appendix S4: Comparison of yearling mass between litters of 2 and 3+ yearlings (no singletons).**

We tested whether the trade-off between yearling mass and number was borne differently, i.e., if the slope of the relationship differed, among yearlings from the same litter in litters of 2-3+ yearlings. We retained only the lightest and the heaviest yearlings in the analysis. When two or more yearlings had the same mass, their relative rank was attributed randomly. We used a linear mixed effects model with maternal age, maternal size (head circumference), litter size (continuous), local population density (relative bear density within a circular buffer around annual female home range centres, see text for details), yearling rank (2 levels: “lightest” or “heaviest”) and the interaction between litter size and yearling rank as explanatory variables and yearling mass (log-transformed to meet model assumption of homoscedasticity) as the response variable. Random effects included litter identity nested in maternal identity and year of capture. The starting model included all fixed effects and we obtained the final model by backwards selection using Likelihood ratio tests. Variance Inflation Factors were all < 3.

**Table S4:** Estimates from a linear mixed effects model to test whether the trade-off between offspring mass and number is borne differently among yearlings from the same litter in litters of 2 and 3+ yearlings (no singletons) in brown bears from south-central Sweden, 1990-2016 (n=192 yearlings).

|  |  |  |  | 95% CI | |
| --- | --- | --- | --- | --- | --- |
| Variables | β | SE | t-value | Lower | Upper |
| ***(b)   Yearling mass – Rank effect*** *(conditional R^2^ = 91%, marginal R^2^ = 34%)** | | | | | |
| Intercept | 0.755 | 0.389 | 1.938 | 0.002 | 1.517 |
| Litter size | -0.023 | 0.040 | -0.581 | -0.101 | 0.058 |
| Maternal size | 0.041 | 0.007 | 6.422 | 0.029 | 0.054 |
| Local density | -0.276 | 0.105 | -2.628 | -0.491 | -0.070 |
| Rank (lightest) | -.156 | 0.067 | 2.331 | 0.025 | 0.288 |
| Litter size × Rank (lightest) | -0.142 | 0.027 | -5.265 | -0.195 | -0.089 |
|  |  |  |  |  |  |
| Random effects | Variance | SD |  |  |  |
| Litter ID × Maternal ID | 0.015 | 0.121 |  |  |  |
| Maternal ID | 0.006 | 0.078 |  |  |  |
| Year | 0.033 | 0.182 |  |  |  |
| Residual | 0.009 | 0.093 |  |  |  |
|  |  |  |  |  |  |
| *Variables excluded: Litter size × Maternal size (χ^2^=1.760, P = 0.185),* *Sex (χ^2^=0.004, P = 0.949), Sex ratio (χ^2^ =0.016, P = 0.890), Maternal age (χ^2^ = 2.122, P = 0.145)* | | | | | |

* Conditional and marginal coefficients of determination (R^2^ [7]) were obtained using the R package MuMIn [8].


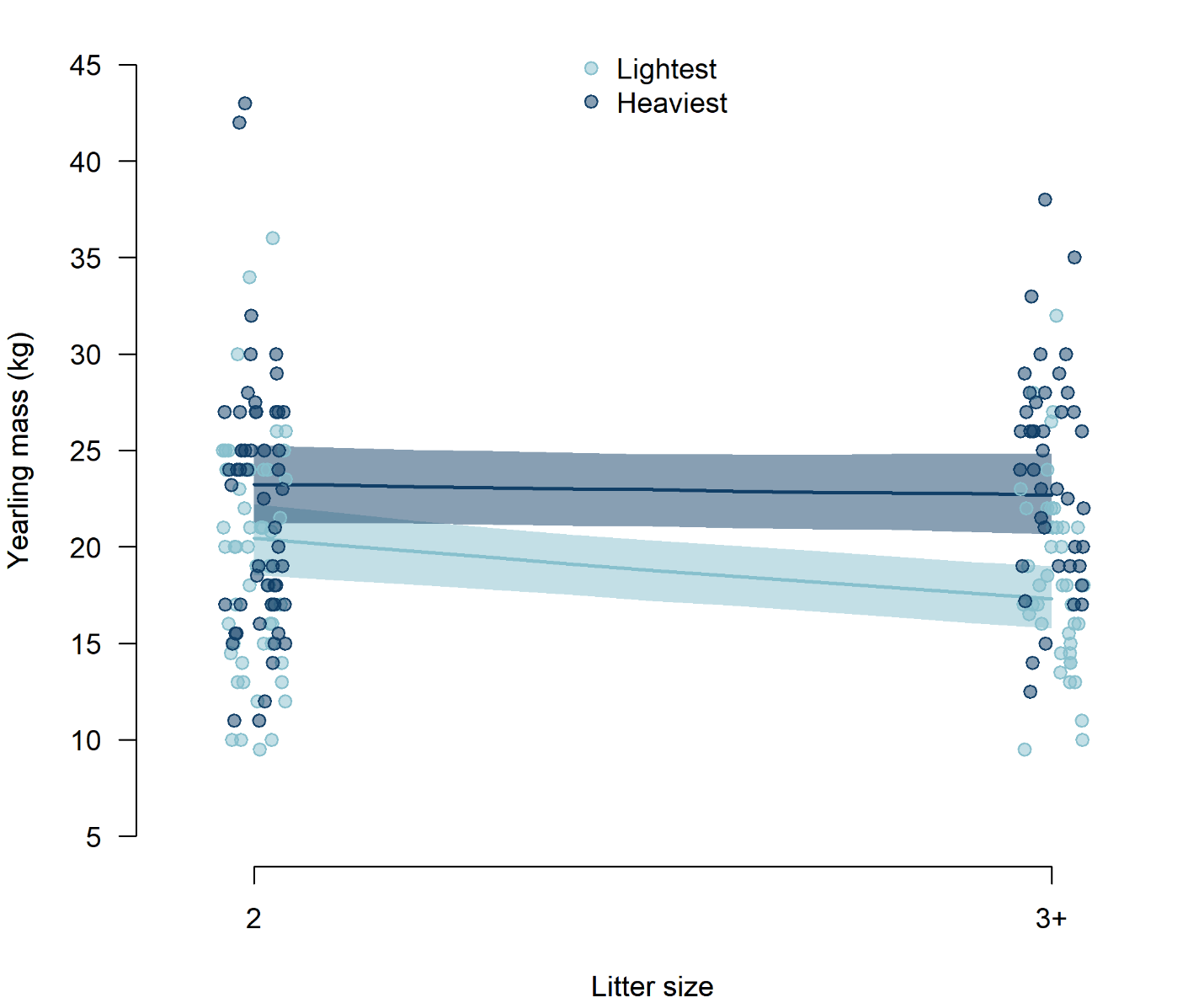


**Figure S1.** Trade-off between offspring mass and number in Scandinavian brown bears, investigated from the perspective of the lightest (light blue) and the heaviest (dark blue) yearling from a litter in litters of 2 and 3+ yearlings (n = 93 litters). The trade-off (negative relationship) between yearling mass and litter size is strongest when considering only the lightest yearling and disappears when considering only the heaviest yearling. In the figure, observations (circles) and model predictions (solid lines) are back-transformed on the original scale for yearlings from average-size mothers at average local density, along with 95 % confidence intervals (shaded polygons).

References

1. Bellemain E, Swenson JE, Tallmon D, Brunberg S, Taberlet P. 2005 Estimating population size of elusive animals with DNA from hunter-collected feces: four methods for brown bears. *Conserv. Biol.* **19**, 150–161. (doi:10.1111/j.1523-1739.2005.00549.x)

2. Kindberg J, Swenson JE, Ericsson G, Bellemain E, Miquel C, Taberlet P. 2011 Estimating population size and trends of the Swedish brown bear *Ursus arctos* population. *Wildlife Biol.* **17**, 114–123. (doi:10.2981/10-100)

3. Leclerc M, Frank SC, Zedrosser A, Swenson JE, Pelletier F. 2017 Hunting promotes spatial reorganization and sexually selected infanticide. *Sci. Rep.* **7**, 45222. (doi:10.1038/srep45222)

4. Swenson JE, Schneider M, Zedrosser A, Söderberg A, Franzén R, Kindberg J. 2017 Challenges of managing a European brown bear population; lessons from Sweden, 1943–2013. *Wildlife Biol.* , doi:10.2981/wlb.00251.

5. Dahle B, Swenson JE. 2003 Seasonal range size in relation to reproductive strategies in brown bears *Ursus arctos*. *J. Anim. Ecol.* **72**, 660–667. (doi:10.1046/j.1365-2656.2003.00737.x)

6. Friebe A, Swenson JE, Sandegren F. 2001 Denning chronology of female brown bears in central Sweden. *Ursus* **12**, 37–45.

7. Nakagawa S, Schielzeth H. 2013 A general and simple method for obtaining R2 from generalized linear mixed-effects models. *Methods Ecol. Evol.* **4**, 133–142. (doi:10.1111/j.2041-210x.2012.00261.x)

8. Barton K. 2009 MuMIn: Multi-model inference. R package version 1.40.0. https://cran.r-project.org/package=MuMIn.
